# How to use BAPV to alleviate the urban heat island effect: An evolutionary game perspective

**DOI:** 10.1371/journal.pone.0296743

**Published:** 2024-01-29

**Authors:** Hongliang Fu, Linye Fu, Haoyu Xie, Xuyi Tian

**Affiliations:** 1 School of Energy and Power Engineering, Shenyang Institute of Engineering, Shenyang, Liaoning, China; 2 School of Energy and Power Engineering, Northeast Electric Power University, Jilin, Jilin, China; 3 School of Electric Power Engineering, Shenyang Institute of Engineering, Shenyang, Liaoning, China; Vardhaman College of Engineering, INDIA

## Abstract

In recent years, the phenomenon of the urban heat island caused by the rapid development of cities is very serious. To solve the problem of the urban heat island, this study proposed a PPP project consisting of the government (GOVT), photovoltaic investment company (PVIC), and residential customers (RS). Based on an evolutionary game model and combined with current policies and industry regulations in China, the evolution process and stable evolution strategies were studied. The result shows that more government subsidies, higher carbon trading prices, and feed-in tariffs will promote the development of the PPP project. For relatively suitable reference value ranges, the installation tilt angle of the BAPV system is 30°, the photovoltaic grid electricity price is 0.1096∼0.1296 $/kWh, the carbon trading is 8.92∼9.42 $/t.

## 1. Introduction

With the use of traditional fossil energy, population agglomeration, energy consumption and greenhouse gas emissions have caused the urban heat island effect and urban climate problems [1]. The phenomenon of the urban heat island refers to the phenomenon that the temperature in urban areas is higher than that in surrounding rural areas, forests and other natural environments [2]. At the beginning of the 20th century, the urban heat island effect gradually attracted the attention of urban planners [3, 4]. At present, the urban heat island effect has become one of the common environmental problems in the world. The urban heat island phenomenon has not only brought many adverse effects to the urban environment, but also brought significant harm in aggravating climate change, increasing air pollution and affecting human health [5, 6].

BAPV is actually a solar photovoltaic power generation system attached to a building, also known as "installed" solar photovoltaic buildings. Its main function is to generate electricity, which does not conflict with the function of the building, and does not destroy or weaken the function of the original building. BAPV can greatly improve the energy efficiency of buildings. It can reduce the radiation of sunlight as much as possible under the premise of ensuring a certain amount of lighting, thus reducing the use of air conditioning in the building in summer and reducing the energy consumption of the building. At the same time, BAPV can also provide functions such as warmth in winter. Therefore, it solves the urban heat island problem to a certain extent. The differences between BAPV and BIPV are as follows: BIPV has been an indispensable part of the building plays the role of building materials, not only can meet the functional requirements of photovoltaic power generation but also can take into account the functional requirements of the building, is a combination of photovoltaic products and building materials, can replace some traditional building materials, integrated design in the building design stage, in the construction and the main body of the building molding. The components in the BAPV building are only attached to the building through a simple support structure, and after removing the photovoltaic modules, the building function remains intact [7, 8]. There are three main methods for monitoring the urban heat island effect: temperature-based heat island monitoring method, vegetation-index-based heat island monitoring method and "thermal landscape"-based heat island monitoring method. Palme et al. [9] studied the problem that can support the calculation of the urban heat island in building performance simulation. The accumulation of heat in the urban space will inevitably lead to the increase of land surface temperature. Therefore, the study of the urban heat island effect based on land surface temperature is one of the most intuitive research methods. By means of remote sensing, scholars have conducted a lot of research on the urban heat island effect. Xie et al. [10] evaluated the potential benefits of photovoltaic pavement to mitigate the urban heat island effect. The result shows that compared with traditional pavement, photovoltaic pavement can reduce the surface temperature by 3–5°C in summer, and reduce the heat generation under various climatic conditions by 11–12%.

Tran et al. [11] compared and evaluated the surface urban heat island of 18 megacities in temperate and tropical climate zones based on Landsat ETM+ data, studied the urban heat island phenomenon in Asia as a whole, and added practical support for the land surface model of high-resolution satellite thermal sensor data; Liang et al. [12] combined weather research, forecasting models, plate models, and single-layer urban canopy models to simulate the urban heat island effect in Beijing, and found that using accurate emissivity can provide the best simulation result. There is a negative correlation between near-surface temperature and emissivity and albedo, while there is a positive correlation between near-surface temperature and urban proportion. Zhang et al. [13] analyzed the relationship between urban land use and six socio-ecological variables and land surface temperature from 2000 to 2013 based on the spatial-temporal change characteristics of land surface temperature in Nanchang. The study showed that the rise of land surface temperature was generally affected by rapid urbanization in the region.

According to the author’s research, in the currently published papers, there is no impact analysis study based on the evolutionary game theory on collaborative projects between government (GOVT), photovoltaic investment company (PVIC) and residential customers (RS). In order to fill this research gap, this study utilized evolutionary game theory, combined it with current policies and industry regulations in China to establish a tripartite evolutionary game model and studied the typical parameters that affect the project, which analyzed the evolution process of the participants, and analyzed and predicted the future development of China to solve the urban heat island problem from the perspective of bounded rationality.

The research of this paper is divided into the following parts: The first chapter introduces the harm of urban heat island effect and expounds the importance of solving urban heat island problem. The second chapter is the mathematical modeling of this research. The third chapter verifies the stability. The fourth chapter analyzes and discusses the modeling results. Chapter five summarizes and looks forward to this study.

## 2. Modeling approach

### 2.1 Problem description

With the continuous increase of PPP (Public-Private-Partnership) projects in recent years, PPP refers to a partnership between the government and private organizations, based on a concession agreement, to provide a certain public good and service, and to clarify the rights and obligations of both parties by signing contracts to ensure the smooth completion of cooperation, and ultimately, achieving a more favorable outcome for all parties involved in the cooperation than expected through individual actions; Under uncontrollable disasters, PPP has also demonstrated its unique stability and trust.

In this project, the main participants are GOVT, PVIC and RS. In the background of promoting zero carbon emissions of the construction industry in China, GOVT will introduce a series of subsidy policies for the construction and utilization of the BAPV system for power generation. GOVT also needs to regulate the stability of the electricity market and carbon emission market, so it will bear part of the financial burden. For PVIC, it mainly pursues profit maximization. It will not only cooperate with RS to build the BAPV system, but also sell the BAPV system to them. In addition, because it has built the BAPV system, PVIC will receive subsidies and rewards from GOVT; For RS, it is a supply-demand relationship with PVIC, and they always hope to obtain more profits and subsidies.

In addition to encouraging PVIC and RS to build the BAPV system, GOVT subsidies for the BAPV system power generation will also encourage the development of the entire photovoltaic construction industry. The development of the photovoltaic construction industry cannot be separated from the profit balance of management stakeholders. Hence, the balance of cooperation projects between the main stakeholders—GOVT, PVIC and RS needs to be focused on. The three main participants have different profit demands, so this study explores the development strategies of the future photovoltaic construction industry by selecting GOVT, PVIC, and RS as the main stakeholders in a tripartite interest study. However, further discussion is needed on the cooperation models of GOVT, PVIC, and RS.

### 2.2 Evolutionary game theory method

In the traditional game theory, it is often assumed that the participants are entirely rational, and the participants are conducted under the condition of complete information. However, for participants in real economic life, it is difficult to achieve the conditions of entire rationality and complete information [14]. In the cooperation and competition of enterprises, there are differences between participants. The complexity of the economic environment and the game itself leads to incomplete information and bounded rationality of participants.

Different from the traditional game theory, the evolutionary game theory does not require people to be entirely rational, nor does it require conditions for complete information [15]. The evolutionary game theory, which is derived from the biological evolution theory, is a theory that combines game theory analysis with dynamic evolution process analysis. In terms of methodology, it is different from the game theory which focuses on static equilibrium and comparative static equilibrium, and emphasizes a dynamic equilibrium [16, 17].

Evolutionary game theory abandons the assumption of complete rationality and takes Darwinian evolution and Lamarck’s genetic theory as the ideological basis. Starting from system theory, it regards the adjustment process of group behavior as a dynamic system, in which each individual’s behavior and the relationship between them and the group are described separately. The formation mechanism from individual behavior to group behavior and various factors involved can be incorporated into the evolutionary game model to form a macro model with micro basis, so as to more truly reflect the diversity and complexity of actors, and provide a theoretical basis for macro-control of group behavior.

### 2.3 Formulas for modeling

At present, BAPV is the mainstream type of photovoltaic construction, which attaches the photovoltaic system to existing buildings. However, its installation and maintenance are more convenient. For the same installation area, the cost of the BAPV system is 30% higher than that of the BIPV system [18–29]. Fang et al. [20] used the cost and recovery value formula to calculate the recovery cost of electric vehicles, which is a general formula, and this study transforms and utilizes it. Therefore, the cost and recovery value formula for solar panels in the BAPV system is as follows.

The cost of solar panels:

$$A=PK\frac{r{\left(1+r\right)}^{L}}{{\left(1+r\right)}^{L}-1}$$ (1)

Among the letters, *P* represents the power of solar panels, *K* is the unit cost of solar panels, *L* represents the service life of solar panels, *r* is the bank rate.

The recycling value of solar panels:

D=PK(1−m) (2)

Among the letters, *D* represents the whole recycling value of solar panels, *m* is the depreciation rate of solar panels,

Programme 1:(1. 1. 1), GOVT, PVIC and RS all participate in the cooperation. GOVT supervises the entire power market and photovoltaic carbon emission trading market. At the same time, it gives incentives to PVIC and RS to set up the BAPV system. PVIC builds the BAPV system to obtain some electricity sales profits, all carbon emission trading profits and subsidies from GOVT. RS agree that PVIC builds the BAPV system and receive profits from electricity sales and subsidies from GOVT.


$$\left\{\begin{array}{l} s_{1}=-C_{1}-GF_{1}-GF_{2}\\ s_{2}=kGi_{1}+GF_{1}+GWi_{2}+D-A\\ s_{3}=(1-k)Gi_{1}+GF_{2} \end{array}\right.$$ (3)

Among the letters, *G* is the annual power generation of the BAPV system,which is simulated by meteorological data, etc.

Programme 2:(0. 1. 1), PVIC and RS participate in the cooperation, but GOVT does not participate in the cooperation. GOVT passively supervises the entire power market and photovoltaic carbon trading market, and will not reward PVIC and RS to build the BAPV system. PVIC builds the BAPV system to obtain some electricity sales profits and all carbon emission trading profits. RS agree that PVIC builds the BAPV system and obtain profits from electricity sales.


$$\left\{\begin{array}{l} s_{1}=-C_{2}\\ s_{2}=kGi_{1}+GWi_{2}+D-A\\ s_{3}=(1-k)Gi_{1} \end{array}\right.$$ (4)

Programme 3:(0. 1. 0), PVIC participates in the cooperation, but GOVT and RS do not participate in the cooperation. GOVT passively supervises the entire power market and photovoltaic carbon trading market, and will not reward PVIC to build the BAPV system. PVIC builds the BAPV system, and obtain all profits from power sales and all profits from carbon emission trading. RS will not receive profits from electricity sales.


$$\left\{\begin{array}{l} s_{1}=-C_{2}\\ s_{2}=Gi_{1}+GWi_{2}+D-A\\ s_{3}=0 \end{array}\right.$$ (5)

Programme 4:(0. 0. 1), RS participate in the cooperation. GOVT and PVIC do not participate in the cooperation. GOVT passively supervises the entire power market and photovoltaic carbon trading market, and will not reward RS for building the BAPV system. PVIC sells the BAPV system to RS to obtain profits from selling photovoltaic panels. RS build the BAPV system and get profits from electricity sales.


$$\left\{\begin{array}{l} s_{1}=-C_{2}\\ s_{2}=A\\ s_{3}=Gi_{1}+D-A \end{array}\right.$$ (6)

Programme 5:(1. 0. 0), GOVT participates in the cooperation, but PVIC and RS do not participate in the cooperation. GOVT actively supervises the entire power market and photovoltaic carbon trading market, and will reward RS for building the BAPV system. PVIC does not sell or build. RS do not buy or build.


$$\left\{\begin{array}{l} s_{1}=-C_{1}\\ s_{2}=0\\ s_{3}=0 \end{array}\right.$$ (7)

Programme 6:(1. 0. 1), GOVT and RS participate in the cooperation, but PVIC does not participate in the cooperation. GOVT actively supervises the entire power market and the photovoltaic carbon trading market, and will reward RS for building the BAPV system. PVIC sells the BAPV system to RS to obtain profits from selling photovoltaic panels. RS build the BAPV system and earn profits from electricity sales.


$$\left\{\begin{array}{l} s_{1}=-C_{1}\\ s_{2}=A\\ s_{3}=Gi_{1}+D-A \end{array}\right.$$ (8)

Programme 7:(1. 1. 0), GOVT and PVIC participate in the cooperation, but RS do not participate in the cooperation. GOVT actively supervises the entire power market and photovoltaic carbon trading market, and will reward PVIC to build the BAPV system. PVIC will build the BAPV system to obtain all the profits from power sales and carbon emission trading. RS will not receive profits from electricity sales.


$$\left\{\begin{array}{l} s_{1}=-C_{1}\\ s_{2}=Gi_{1}+GWi_{2}+D-A\\ s_{3}=0 \end{array}\right.$$ (9)

Programme 8:(0. 0. 0), GOVT, PVIC and RS do not participate in the cooperation. GOVT passively supervises the entire power market and photovoltaic carbon emission trading market, and will not reward RS for building the BAPV system. PVIC does not sell or build. RS does not buy or build.


$$\left\{\begin{array}{l} s_{1}=-C_{2}\\ s_{2}=0\\ s_{3}=0 \end{array}\right.$$ (10)

The core element of the evolutionary game is the evolutionary stability strategy (ESS), which reflects the dynamic convergence process from the starting point to the steady state. The dynamic equations of replicate factors are used to describe the steady state of the evolutionary game. The dynamic equations of replicate factors derived from the above eight sets of income matrices are:

$$f_{\text{GOVT}}(x)=\frac{\text{d}x}{\text{d}t}=x(1-x)\left\{\left[-G(F_{1}+F_{2})\right]yz-C_{1}-C_{2}\right\}$$ (11)

$$f_{\text{PVIC}}(y)=\frac{\text{dy}}{\text{d}t}=y(1-y)\left\{GF_{1}xz+(k-1)Gi_{1}z+Gi_{1}+GWi_{2}+D-A\right\}$$ (12)

$$f_{\text{RC}}(z)=\frac{\text{dz}}{\text{d}t}=z(1-z)\left\{GF_{2}xy-(kGi_{1}+D-A)y+Gi_{1}+D-A\right\}$$ (13)

From the dynamic equations of replicate factors, it can be seen that the different cooperation methods of GOVT, PVIC and RS will have different impacts on their interests. When the expected benefits are better than the average benefits, the proportion will increase.

## 3. Results and discussion

### 3.1 Assumptions and initial parameters

For the tripartite evolutionary game model in this study, there are mainly the following assumptions:

1. People are facing a complex and uncertain world, and the more transactions there are, the greater the uncertainty and the less complete the information; People’s abilities to calculate and understand the environment are limited, and people cannot know everything. Therefore, the three participants in the model are bounded rationality and non-information symmetry.
2. GOVT’s strategies are to actively regulate the power market and photovoltaic carbon emission trading market and issue subsidies, or passively regulate the power market and photovoltaic carbon emission trading market and not issue subsidies; The strategies of PVIC include investing and installing the BAPV system, or selling the BAPV system to RS; RS’ strategies include collaborating with PVIC to build the BAPV system, or purchasing their own BAPV system to build.

In order to alleviate the problem of the urban heat island, the Chinese government has proposed a series of policies to promote the development of the building photovoltaic industry. In this context, this paper studies the collaborative development of PVIC and RS under the promotion of the Chinese government to promote the development of China’s construction photovoltaic industry based on the evolutionary game. Therefore, the initial parameters of the evolutionary game simulation in this paper are determined in accordance with the current relevant policies and industry regulations in China, as shown in Table 1 in S1 Appendix.

For the BAPV system proposed in this study, assuming the total power of the BAPV system is 45KW, for the government, it has the expenditure cost of *C*_1_ = 0.5×10^8^ $ to actively regulate the market and *C*_2_ = 0.1×10^8^ $ to passively regulate the market [21]. PVIC, mainly builds and sells the BAPV system, so it will focus on the photovoltaic grid price, photovoltaic power generation subsidy, and carbon emission trading price. Through literature review, Ding et al. [22] listed the PV subsidy standard of PVIC in Shenzhen as 0.3 yuan /kWh. Therefore, this study assumed that the above three indicators were *i*_1_ = 0.1096 $/kWh, *i*_2_ = 8.92 $/t, *F*_1_ = 0.0429 $/kWh. For the BAPV system, ased on the economic and environmental benefit analysis data of the BAPV system in Shanghai conducted by Wang et al. [23], this study assumes that the unit cost is 1947.4 $/kWh, and each degree of electricity can reduce carbon dioxide emissions by 12.43×10^−6^ t/kWh. It has a service life of 25 years and a depreciation rate of 3%. For RS, its main expenses are the cost of purchasing the BAPV system and getting subsidies from GOVT. Taking Shenzhen City as an example, *F*_2_ = 0.0571 $/kWh can be obtained [24–26].

### 3.2 Stability analysis of evolutionary games

The Liapunov discriminant method is used to analyze the stability of the tripartite evolutionary game theory. When all eigenvalues of the Jacobian matrix are negative, the model can be considered stable [27–29].

The Jacobian matrix of this study is as follows:

$$J=\left[\begin{array}{ccc} \frac{\partial f_{\text{govt}}(x)}{\partial x} & \frac{\partial f_{\text{govt}}(x)}{\partial y} & \frac{\partial f_{\text{govt}}(x)}{\partial z}\\ \frac{\partial f_{\text{pic}}(y)}{\partial x} & \frac{\partial f_{\text{pic}}(y)}{\partial y} & \frac{\partial f_{\text{pic}}(y)}{\partial z}\\ \frac{\partial f_{\text{rs}}(z)}{\partial x} & \frac{\partial f_{\text{rs}}(z)}{\partial y} & \frac{\partial f_{\text{rs}}(z)}{\partial z} \end{array}\right]$$ (14)

By setting *f*_govt_ = 0,*f*_pvic_ = 0 and *f*_rs_ = 0,we can get eight pure strategy solutions for this evolutionary game model, that is (1, 1, 1), (1, 1, 0), (1, 0, 1), (0, 1, 1), (0, 0, 1), (0, 1, 0), (1, 0, 0), (0, 0, 0). Meanwhile, the eight stable points form the boundary of the evolutionary game, that is {(*x*,*y*,*z*)0 ≤ *x* ≤ 1;0 ≤ *y* ≤ 1;0 ≤ *z* ≤ 1}. Besides, there are other mixed strategy solutions in the evolutionary game system, but they are not ESS points. Therefore, this paper focuses on studying the ESS points in the eight pure strategy situations mentioned above.

According to Lyapunov stability theory, when all eigenvalues of the Jacobian matrix of the system are negative, the equilibrium point gradually becomes stable, otherwise, it is not a stable point. First, the eight equilibrium points are substituted into the Jacobian matrix, and then the positive and negative forms are judged. Then, whether the equilibrium points are the ESS of the system is judged, and the conditions for forming the ESS are determined. Through derivation of the income matrix in the previous chapter, different eigenvalues in eight cases are obtained. According to Lyapunov stability theory, ESS points of this mathematical model can be obtained, as shown below:

For the tripartite cooperation between GOVT, PVIC and RS (1. 1. 1) mode, the eigenvalues are: The eigenvalues for (1. 1. 1) cooperation mode are:

$$\left\{\begin{array}{l} \lambda_{1}=G(F_{1}+F_{2})+C_{1}+C_{2}\\ \lambda_{2}=-GF_{1}-kGi_{1}-GWi_{2}-D+A\\ \lambda_{3}=-GF_{2}+kGi_{1}-Gi_{1} \end{array}\right.$$ (15)

For the mode (1. 1. 0) in which GOVT and PVIC participate but RS does not, the eigenvalues are: The eigenvalues for (1. 1. 0) cooperation mode are:

$$\left\{\begin{array}{l} \lambda_{1}=C_{1}+C_{2}\\ \lambda_{2}=-Gi_{1}-GWi_{2}-D+A\\ \lambda_{3}=GF_{2}-kGi_{1}-Gi_{1} \end{array}\right.$$ (16)

For the mode (1. 0. 1) in which GOVT and RS participate but PVIC does not, the eigenvalues are: The eigenvalues for (1. 0. 1) cooperation mode are:

$$\left\{\begin{array}{l} \lambda_{1}=C_{1}+C_{2}\\ \lambda_{2}=GF_{1}+kGi_{1}+GWi_{2}+D-A\\ \lambda_{3}=-Gi_{1}-D+A \end{array}\right.$$ (17)

For the mode (0. 1. 1) in which RS and PVIC participate in cooperation but GOVT does not, the eigenvalues are: The eigenvalues for (0. 1. 1) cooperation mode are:

$$\left\{\begin{array}{l} \lambda_{1}=-G(F_{1}+F_{2})-C_{1}-C_{2}\\ \lambda_{2}=-kGi_{1}-GWi_{2}-D+A\\ \lambda_{3}=kGi_{1}-Gi_{1} \end{array}\right.$$ (18)

For the mode (0. 1. 0) in which PVIC participates in cooperation while RS and GOVT do not, the eigenvalues are: The eigenvalues for (0. 1. 0) cooperation mode are:

$$\left\{\begin{array}{l} \lambda_{1}=-C_{1}-C_{2}\\ \lambda_{2}=-Gi_{1}-GWi_{2}-D+A\\ \lambda_{3}=-kGi_{1}+Gi_{1} \end{array}\right.$$ (19)

For the mode (0. 0. 1) in which RS participates in cooperation while PVIC and GOVT do not, the eigenvalues are: The eigenvalues for (0. 0. 1) cooperation mode are:

$$\left\{\begin{array}{l} \lambda_{1}=-C_{1}-C_{2}\\ \lambda_{2}=GWi_{2}+D-A\\ \lambda_{3}=-Gi_{1}-D+A \end{array}\right.$$ (20)

For the mode (1. 0. 0) in which GOVT participates in cooperation while PVIC and RS do not, the eigenvalues are: The eigenvalues for (1. 0. 0) cooperation mode are:

$$\left\{\begin{array}{l} \lambda_{1}=C_{1}+C_{2}\\ \lambda_{2}=Gi_{1}+GWi_{2}+D-A\\ \lambda_{3}=Gi_{1}+D-A \end{array}\right.$$ (21)

For the mode (0. 0. 0) in which GOVT, PVIC and RS do not participate in the cooperation, the eigenvalues are: The eigenvalues for (0. 0. 0) cooperation mode are:

$$\left\{\begin{array}{l} \lambda_{1}=-C_{1}-C_{2}\\ \lambda_{2}=Gi_{1}+GWi_{2}+D-A\\ \lambda_{3}=Gi_{1}+D-A \end{array}\right.$$ (22)

Based on the eigenvalues and related parameters of the eight cooperation modes, the results of the equilibrium point can be calculated, as shown in Table 2 in S1 Appendix.

From Table 2 in S1 Appendix, it can be seen that among the eight equilibrium points, only (1. 1. 1), (1. 1. 0), and (0. 1. 1) satisfy the Lyapunov stability theory. In addition, the eight equilibrium states are affected by the annual power generation of the BAPV system and cannot be determined as positive or negative. Therefore, the annual power generation of the BAPV system will be simulated in a real environment.

## 4. Simulation analysis

### 4.1 Project introduction

This project is built in a residential building in Beijing, with a length of 30 meters and a width of 24 meters. Therefore, it has a large area for laying photovoltaic modules. The installed capacity of the BAPV system is 45 kW, and the power generation method is fully connected to the grid. It is assumed that it can operate for 25 years.

### 4.2 Effect analyses of typical parameters

In this section, the impact of several typical parameters on the willingness of PVIC and RS to cooperate is studied, including the BAPV system installation inclination, photovoltaic grid price, carbon emission trading price, photovoltaic subsidy, and other parameters. And the impact under the E1 (1. 1. 1) situation is mainly studied.

#### 4.2.1 BAPV system installation inclination

**Fig 1:**
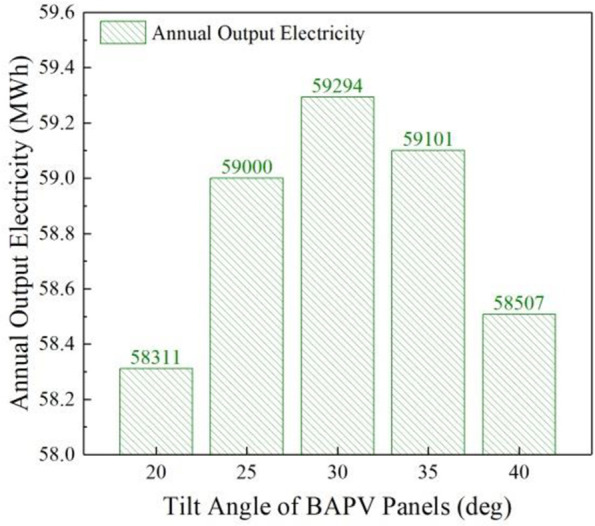
Installation tilt angle α the impact on the annual power generation of the BAPV system.

To build a high-quality BAPV system, attention should be paid to the tilt angle of the installation of solar modules. Therefore, the tilt angle of the installation of solar modules should be adjusted according to different longitude and latitude to obtain the maximum solar radiation and maximize the power generation efficiency of the BAPV system. At the same time, the installation tilt angle determines the annual power generation of the BAPV system, so it is particularly important to explore the installation angle of the BAPV system. Fig 1 shows the specific process.

When the installation tilt angle of the BAPV system is 30°, the annual power generation of the BAPV system is the highest, at 59294 kW; when the installation tilt angle is 20°, the annual power generation of the system is the lowest. The power generation of the system tends to increase first and then decrease as the installation tilt angle increases. This may be due to the difference in the incidence angle of the sun on the inclined surface of the solar panel, which results in a different amount of solar normal radiation received per unit area. The larger the incidence angle, the less solar normal radiation received, and the change in the inclination angle of the solar panel will cause a change in the incidence angle of the sun, thereby affecting its radiation reception, details can be seen in supplementary documents.

**Fig 2:**
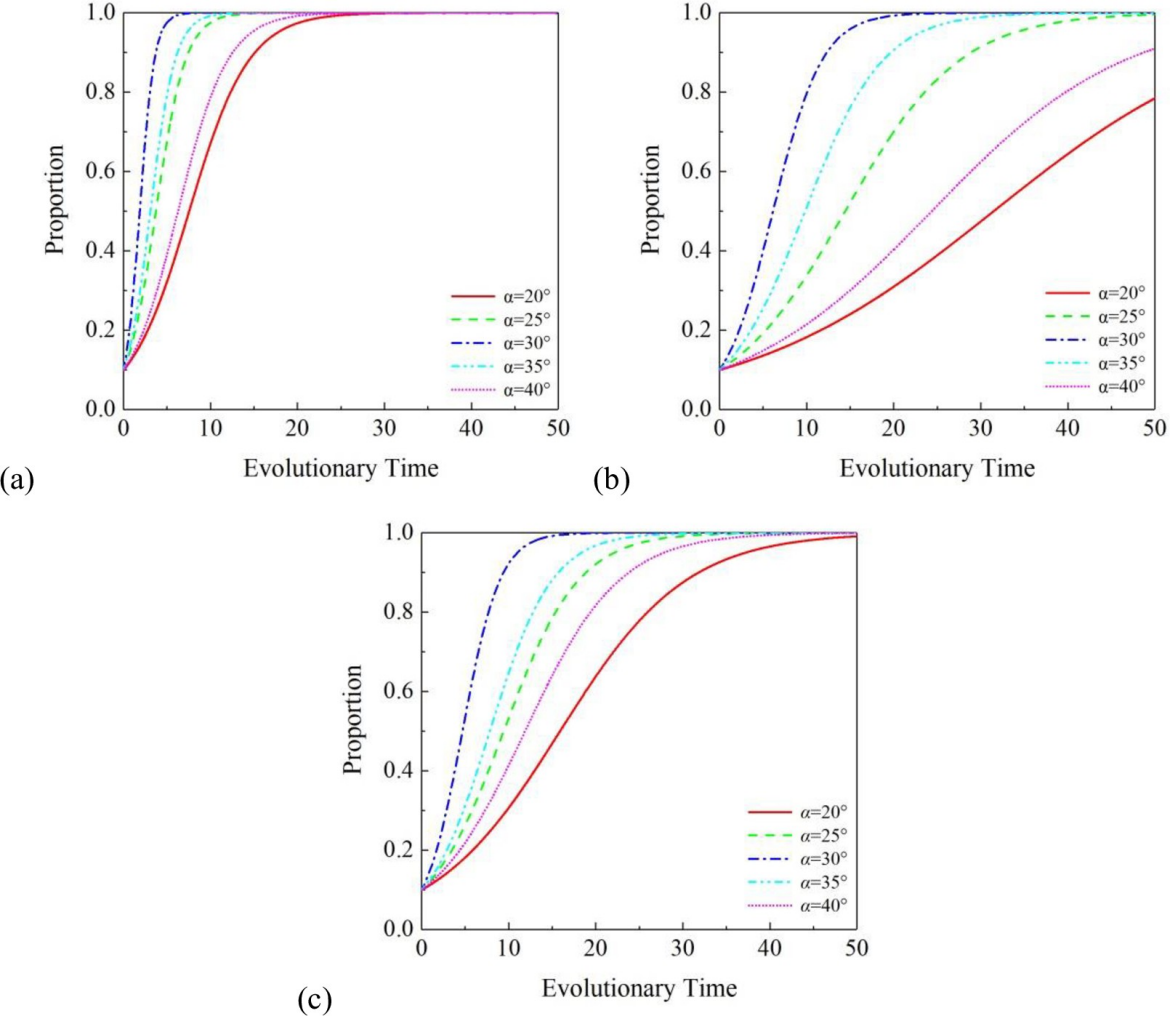
Effects of α under the partnership mode of (1, 1, 1): (a) on GOVT, (b) on PVIC and (c) on RS.

Fig 2 shows the specific process. It can be seen that the installation angle of the BAPV system has a significant impact on the annual power generation of the system, thus having a significant impact on the three participants. When the installation angle is 30°, the growth rate of cooperation willingness of the three participants is the highest. As the installation angle changes from 20° to 40°, the growth rate of cooperation willingness of the three participants also increases first and then decreases. For GOVT, the more power generation, the more subsidies it will spend, but its willingness to cooperate is the strongest. This may be because compared with subsidies, GOVT is more willing to build a BAPV system to slow down the urban heat island phenomenon. From the graph, it can be seen that all three participants tend to collaborate, which to some extent demonstrates the superiority of the project.

Based on the annual power generation and the willingness of the three participants to cooperate, this study selected the installation angle of the BAPV system at 30°, with an annual power generation of 59294 kW.

#### 4.2.2 Photovoltaic grid electricity price

**Fig 3:**
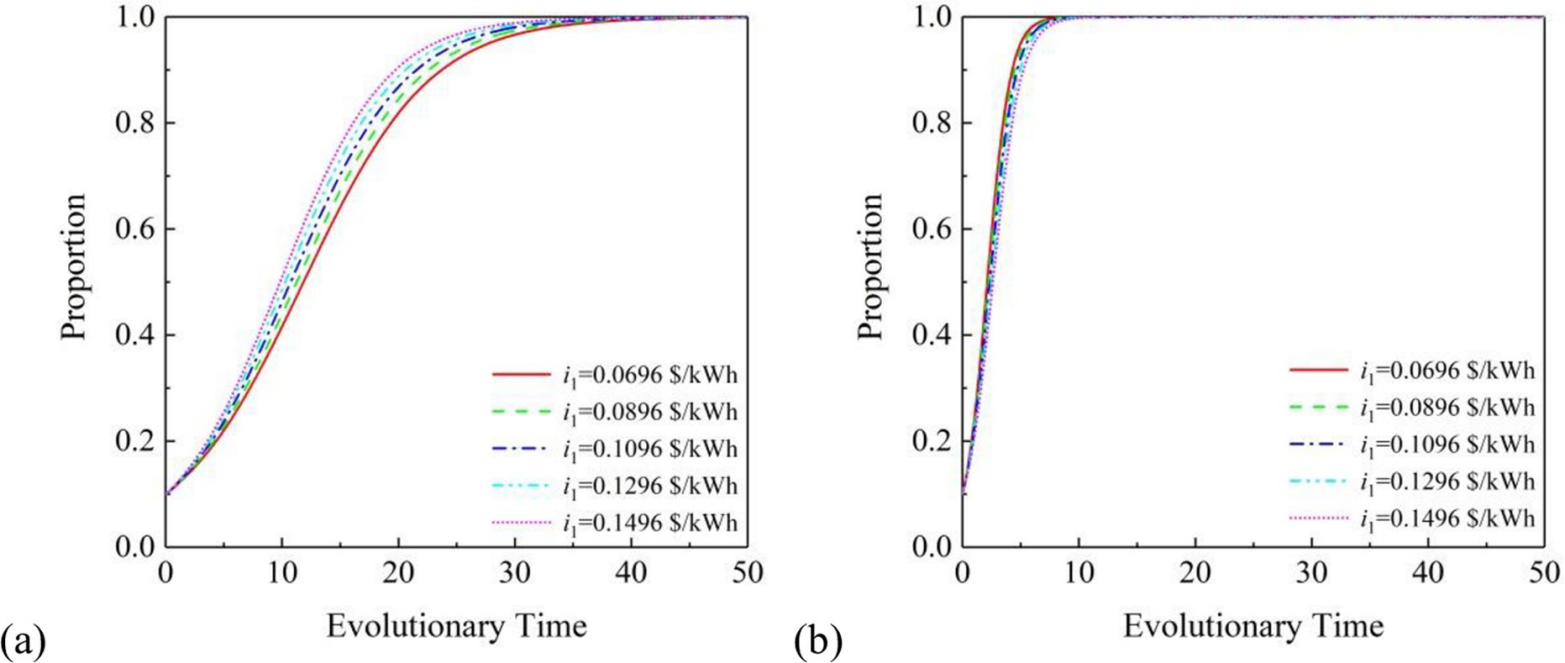
Effects of *i*_1_ under the partnership mode of (1, 1, 1): (a) on PVIC and (b) on RS.

Fig 3 shows the specific process. The photovoltaic grid electricity price is the main source of profit for PVIC and RC, so *i*_1_ has a significant impact on the willingness of PVIC and RS to cooperate. The figure shows the impact of photovoltaic grid price *i*_1_ on the willingness to cooperate between PVIC and RS under the (1. 1. 1) cooperation mode. The results indicate that the impact of photovoltaic grid price on the willingness to participate in PVIC and RS is not significant. With the increase of photovoltaic grid electricity price from 0.0696 $/kWh to 0.1496 $/kWh, the willingness of PVIC and RS to cooperate has also increased. The photovoltaic grid electricity price has a positive impact on the willingness of PVIC and RS to participate, and it can be seen that RS’ willingness to participate in cooperative projects increases faster than PVIC’s willingness to participate, which also indicates the superiority of this project.

#### 4.2.3 Carbon trading prices

**Fig 4:**
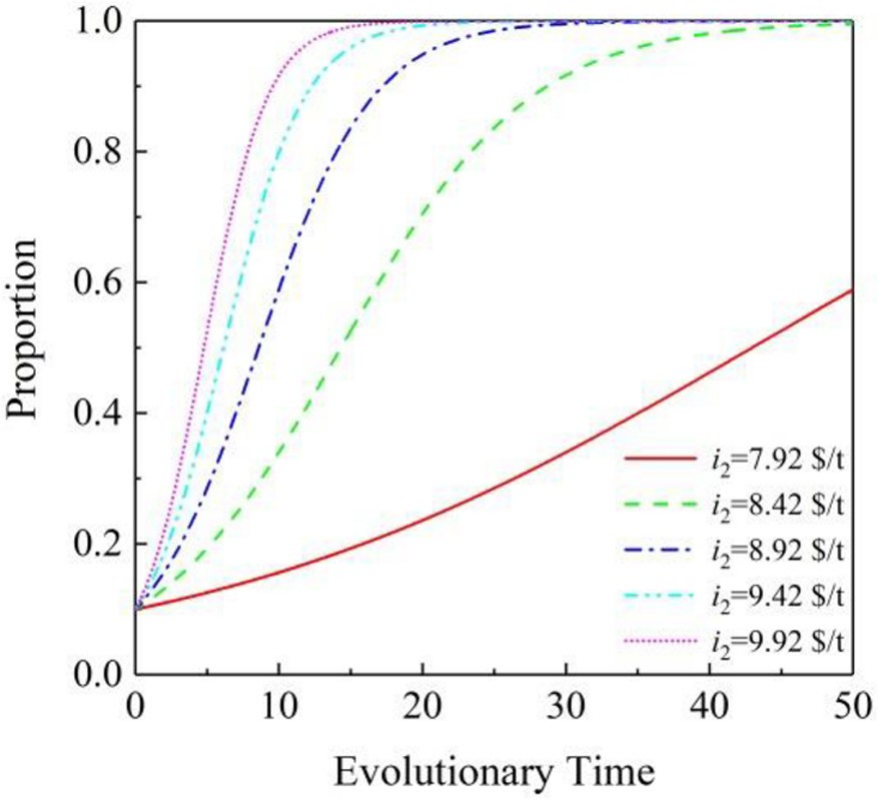
Effects of *i*_2_ under the partnership mode of (1. 1. 1): PVIC.

Fig 4 shows the specific process. Carbon trading is a greenhouse gas emission reduction trade based on international public law in order to promote global greenhouse gas emission reduction under the Kyoto Protocol. Among the six greenhouse gases that are required to be reduced, carbon dioxide (CO_2_) is the largest emission among them. Therefore, this transaction is calculated in the unit of per ton carbon dioxide equivalent (tCO_2_e), so it is generally called "carbon trading". Its trading market is called the carbon market. Therefore, carbon trading price is very important for stabilizing the carbon market.

The figure shows the impact of carbon trading prices on PVIC. The result shows that with the increase in carbon trading price, PVIC’s willingness to cooperate also increases. When *i*_2_ is 7.92 $/t, PVIC will gradually join the cooperation. When *i*_2_ increases to 8.42 $/t, its willingness to cooperate will increase faster. The reasonable carbon trading price can promote the development of the project. For the carbon trading price in this study, it can be set to 8.92 $/t or higher.

#### 4.2.4 Photovoltaic subsidies

**Fig 5:**
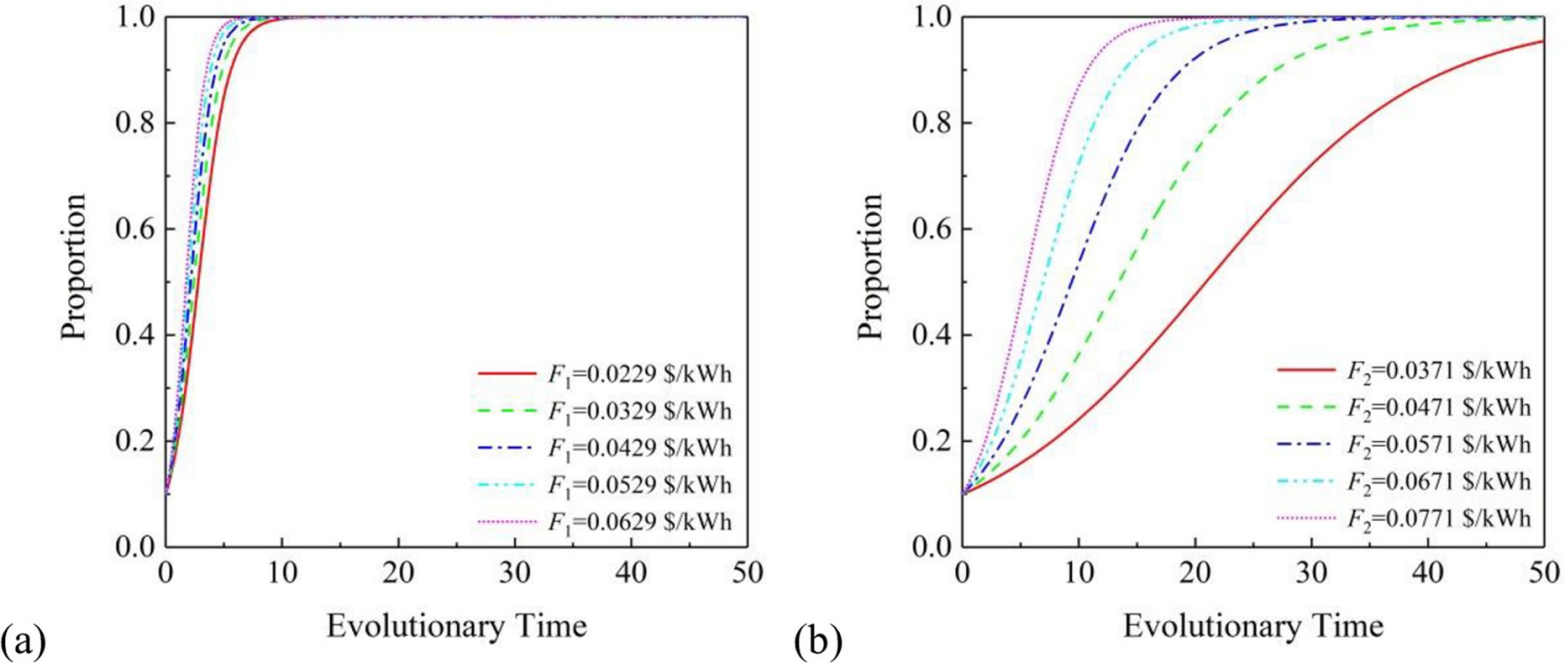
Effects of F under the partnership mode of (1. 1. 1): (a) on PVIC and (b) on RS.

Fig 5 shows the specific process. GOVT subsidies are usually beneficial for PPP projects. To investigate the impact of GOVT subsidies on PVIC and RS in the PPP projects proposed in this chapter, relevant simulation calculations were conducted in this section, and the calculation results are shown in the figure. From the graph, it can be seen that under the cooperation model of (1. 1. 1), the impact of GOVT subsidies on PVIC is not as significant as on RS. With GOVT subsidies increasing from 0.0229 $/kWh to 0.0629 $/kWh at regular intervals, there has been little change in PVIC’s willingness to cooperate, but both have maintained a positive growth rate of willingness to cooperate. Overall, increasing GOVT subsidies can significantly enhance RS’ willingness to cooperate, which means that increasing GOVT subsidies can promote the participation of RS in collaborative development projects and effectively promote the development of the proposed PPP projects.

## 5. Summary and conclusions

In the context of alleviating the problem of urban heat islands, this project proposes a PPP project consisting of GOVT, PVIC and RS. Based on the tripartite evolutionary game and China’s current policies and industry regulations, a simulation was conducted on the evolutionary stability strategy. According to the simulation results, the conclusions can be drawn as follows:

For GOVT, when the installation angle is 30°, its willingness to cooperate is strongest, which may be due to more power generation leads to its participation in cooperation; when the installation angle is 40°, due to more shadows lead to the reduction of its power generation, so its willingness to cooperate is not as good as when the installation angle is 30°. Consequently, the recommended installation angle is 30°.

For PVIC, the parameters that have a greater impact are the carbon trading prices and the installation angle of the BAPV system. The cooperation motivation of PVIC is the total profit that PVIC can obtain. The installation angle of the BAPV system will affect the annual power generation of the system, thereby directly affecting the final profit of the PVIC. And the cost of BAPV devices also affects the initial total investment, so PVIC should pay more attention to its own transformation and upgrading to obtain a system with lower costs and higher power generation. It is obvious that when the installation angle is 30°, PVIC’s willingness to cooperate is strongest. However, when the installation angle is 40°, the willingness is declined, so the recommended installation is 30°.

For RS, the parameters that have a significant impact are the installation inclination and the GOVT subsidies. It is easy to understand, for more GOVT subsidies will provide RS with more additional income, and installation inclination will directly affect the electricity sold by RS. Therefore, these two key influencing factors constrain RS’ willingness to participate in cooperative projects. In order to ensure the stable operation of the project, GOVT should further promote subsidies for RS. The situations are the same as GOVT and PVIC, that is, the installation angle of 30° is the best choice.

All the analysis results in this study can provide a certain theoretical basis for the Chinese government and related industries in formulating policies and relevant industry regulations. In addition, the mathematical model constructed in this study is applicable to similar problems in other countries and regions to a certain extent.

This study also has certain limitations, such as not considering the simultaneous application of BAPV and BIPV in the electricity market. Therefore, based on more complex coupled models is the next research direction.

The limitations of this paper are as follows: first, the influence results under different cooperation modes are not taken into account; second, the game between different brand parties has not been considered; third, the game under the complex coupling of internal and external conditions remains to be studied.

## Supporting information

S1 Appendix (DOCX)

S1 File (ZIP)

## Abbreviations

BAPV: building attached photovoltaic
BIPV: building integrated photovoltaic
GOVT: government
ESS: evolutionary stable strategy
PPP: public-private partnership
PVIC: photovoltaic investment company
RS: residential customers
**Nomenclature**
A: The cost of solar panels ($)
*C*_1_: The cost of actively supervising ($)
C_2_: The cost of passively supervising ($)
D: The total recycling value of solar panels ($)
G: The annual power generation of the system (kW∙year^-1^)
*i*_1_: Photovoltaic grid electricity price ($∙kWh^-1^)
*i*_2_: Carbon trading price ($∙t^-1^)
k: Profit distribution ratio (-)
K: The unit cost of solar panels ($∙W^-1^)
L: The service life of solar panels (year)
m: The depreciation rate of solar panels (-)
P: The power of solar panels (kW)
W: KWh electricity reduction (t∙kWh^-1^)
r: bank rate (-)
**Subscripts**
GOVT: government
PVIC: photovoltaic investment company
RS: residential customers
