# Supplementary material for: How to use BAPV to alleviate the urban heat island effect: An evolutionary game perspective
Source: PLoS One. 2024 Jan 29;19(1):e0296743. doi: 10.1371/journal.pone.0296743 (PMC10824433; doi:10.1371/journal.pone.0296743)
Supplement: S1 Appendix — (DOCX) [file pone.0296743.s001.docx]

# Appendix

**Table 1.** **Evolutionary Game Computing Related Initial Parameters**

| **Participant** | **Parameter** | **Meaning** | **Number** |
| --- | --- | --- | --- |
| GOVT | *C*_1_ | The cost of actively supervising | 0.5×10^8^ $ |
|  | *C*_2_ | The cost of passively supervising | 0.1×10^8^ $ |
| PVIC | *P* | Total power of photovoltaic panels | 45.0 kW |
|  | *i*_1_ | Photovoltaic grid electricity price | 0.1096 $/kWh |
|  | *i*_2_ | Carbon trading price | 8.92 $/t |
|  | *k* | Profit distribution ratio | 80 % |
|  | *F*_1_ | Photovoltaic subsidies | 0.0429 $/kWh |
|  | *W* | KWh electricity reduction | 1.243×10^-7^ t/kWh |
|  | *L* | Service life of solar panels | 25 years |
|  | *r* | bank rate | 5 % |
|  | *m* | Depreciation rate of photovoltaic panels | 3 % |
|  | *K* | The cost of photovoltaic panels | 1947.4 $/kW |
| RS | *F*_2_ | Photovoltaic subsidies | 0.0571 $/kWh |

**Table 2.** **Eigenvalue Results for Equilibrium Points**

| **Equilibrium point** | ***λ*_1_** | ***λ*_2_** | ***λ*_3_** | **Result** |
| --- | --- | --- | --- | --- |
| **E_1_(1. 1. 1)** | - | - | - | ESS |
| **E_2_(1. 1, 0)** | - | - | - | ESS |
| **E_3_(1. 0. 1)** | - | unknown | unknown |  |
| **E_4_(0. 1. 1)** | - | - | - | ESS |
| **E_5_(1. 0. 0)** | - | unknown | unknown |  |
| **E_6_(0. 0. 1)** | unknown | unknown | - |  |
| **E_7_(0. 1. 0)** | unknown | - | + |  |
| **E_8_(0. 0. 0)** | unknown | unknown | unknown |  |
